# Minute-scale detection of SARS-CoV-2 using a low-cost biosensor composed of pencil graphite electrodes

**DOI:** 10.1073/pnas.2106724118

**Published:** 2021-07-09

**Authors:** Lucas F. de Lima, André L. Ferreira, Marcelo D. T. Torres, William R. de Araujo, Cesar de la Fuente-Nunez

**Affiliations:** ^a^Machine Biology Group, Departments of Psychiatry and Microbiology, University of Pennsylvania, Philadelphia, PA 19104;; ^b^Institute for Biomedical Informatics, Perelman School of Medicine, University of Pennsylvania, Philadelphia, PA 19104;; ^c^Institute for Translational Medicine and Therapeutics, Perelman School of Medicine, University of Pennsylvania, Philadelphia, PA 19104;; ^d^Department of Bioengineering, School of Engineering and Applied Science, University of Pennsylvania, Philadelphia, PA 19104;; ^e^Department of Chemical and Biomolecular Engineering, School of Engineering and Applied Science, University of Pennsylvania, Philadelphia, PA 19104;; ^f^Penn Institute for Computational Science, University of Pennsylvania, Philadelphia, PA 19104;; ^g^Portable Chemical Sensors Lab, Department of Analytical Chemistry, Institute of Chemistry, State University of Campinas, São Paulo 13083-970, Brazil

**Keywords:** COVID-19, sensor, point of care, graphite, ACE2

## Abstract

COVID-19 has led to over 3.47 million deaths worldwide and continues to devastate primarily middle- and low-income countries. High-frequency testing has been proposed as a potential solution to prevent outbreaks. However, current tests are not sufficiently low-cost, rapid, or scalable to enable broad COVID-19 testing. Here, we describe LEAD (Low-cost Electrochemical Advanced Diagnostic), a diagnostic test that detects severe acute respiratory syndrome coronavirus 2 (SARS-CoV-2) within 6.5 min and costs $1.50 per unit to produce using easily accessible and commercially available materials. LEAD is highly sensitive toward SARS-CoV-2 spike protein (limit of detection = 229 fg⋅mL^−1^) and displays an excellent performance profile using clinical saliva (100.0% sensitivity, 100.0% specificity, and 100.0% accuracy) and nasopharyngeal/oropharyngeal (88.7% sensitivity, 86.0% specificity, and 87.4% accuracy) samples. No cross-reactivity was detected with other coronavirus or influenza strains. Importantly, LEAD also successfully diagnosed the highly contagious SARS-CoV-2 B.1.1.7 UK variant. The device presents high reproducibility under all conditions tested and preserves its original sensitivity for 5 d when stored at 4 °C in phosphate-buffered saline. Our low-cost and do-it-yourself technology opens new avenues to facilitate high-frequency testing and access to much-needed diagnostic tests in resource-limited settings and low-income communities.

## Significance

High-frequency testing is urgently needed to help prevent the spread of COVID-19. Here, we introduce Low-cost Electrochemical Advanced Diagnostic (LEAD), a diagnostic test that detects SARS-CoV-2 within 6.5 min, costs $1.50 per unit, and uses easily assembled materials such as human angiotensin-converting enzyme 2, modified graphite leads, and a plastic vial. LEAD presents sensitivity comparable to the gold-standard methods (limit of detection for the viral spike protein = 229 fg⋅mL^-1^) and displays an excellent performance profile when tested using clinical saliva (100.0% sensitivity, 100.0% specificity, 100.0% accuracy) and nasopharyngeal/oropharyngeal (88.7% sensitivity, 86.0% specificity, 87.4% accuracy) samples. Finally, no cross-reactivity with other viruses was detected and the test displayed a viable shelf-life of 5 d when stored at 4 °C.

Since its onset, the COVID-19 pandemic has led to 3.47 million deaths and costed our economy billions of dollars (1, 2). Different methods using RT-PCR and serology tests have been used to diagnose SARS-CoV-2 in saliva and nasopharyngeal/oropharyngeal (NP/OP) swab samples (3). RT-PCR has served as the gold-standard test during the pandemic because of its high accuracy and specificity. However, this technique is time-intensive, costly, and requires highly skilled workers for operation, thus making it an unsuitable solution for mass testing, especially in developing countries where resources are limited (4, 5).

Most rapid tests commercially available for diagnosing COVID-19 are antigen-based and exhibit low sensitivity, which limits their ability to accurately identify asymptomatic individuals (6–8), thus hindering proper control of viral spread (6–8). Therefore, there is an urgent need to develop low-cost, sensitive, and rapid tests for the early diagnosis of severe acute respiratory syndrome coronavirus 2 (SARS-CoV-2) infections. In an attempt to overcome such limitations, several portable devices have been developed (9–11) offering rapid and decentralized clinical diagnosis (12–15). However, these electrochemical, colorimetric, serological, and molecular-based technologies are limited by their relatively high cost (>$10 per test), need for materials and equipment that are not easily accessible, requirement of specialized labor, and the fact that they are time-consuming and not sufficiently rapid.

**Fig. 1.**
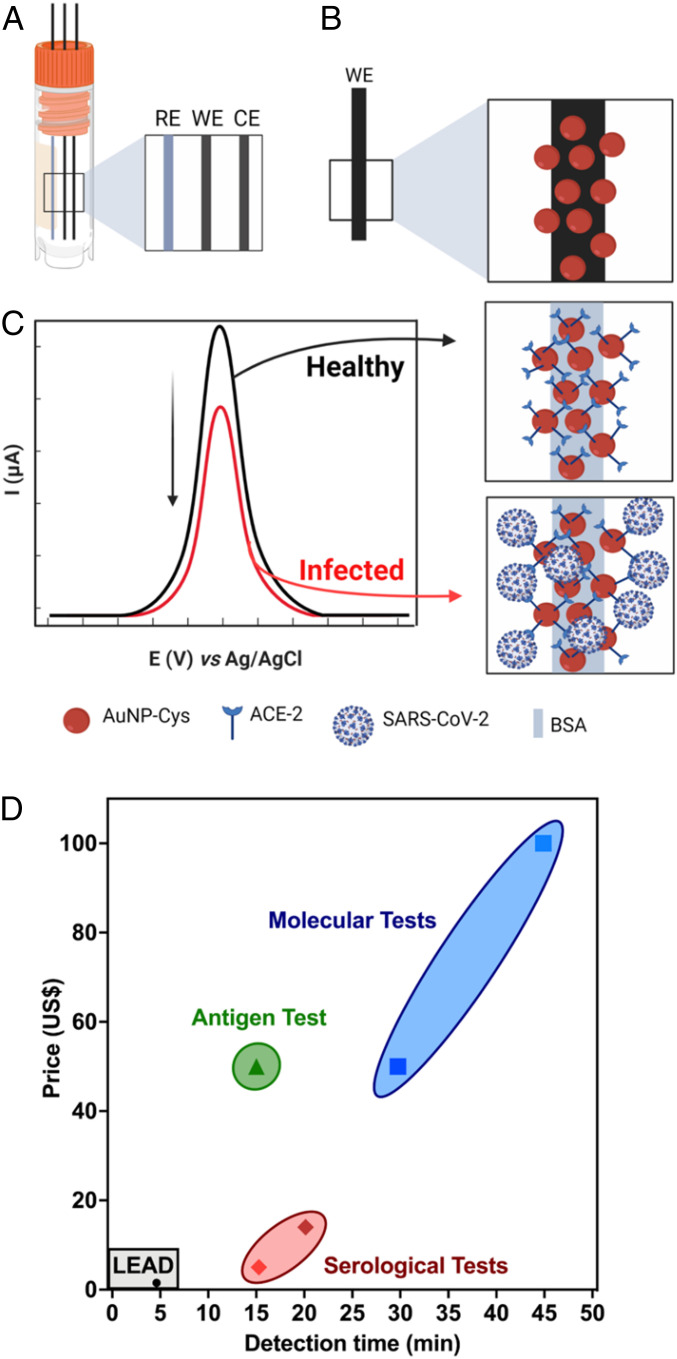
LEAD, a rapid and low-cost electrochemical biosensor. (*A*) Schematic representation of graphite electrodes used in LEAD. (*B*) AuNPs-cys functionalization on graphite electrodes after modification with glutaraldehyde. (*C*) Modification of AuNPs-cys with ACE2 using EDC and NHS to enable amide bond formation and BSA for surface blockage. The analytical response of LEAD in the presence of SARS-CoV-2 was based on current suppression due to selective binding of viral SP with the ACE2-functionalized electrode, which partially blocked the electrodic area, leading to decreased peak current of the redox probe ([Fe(CN)_6_]^3−/4−^ solution). (*D*) Cost and detection time comparison between LEAD and existing FDA-approved antigen, serological, and molecular tests (47).

Here, we report LEAD (Low-cost Electrochemical Advanced Diagnostic), a technology for rapid COVID-19 diagnosis that uses highly accessible and inexpensive materials. The do-it-yourself (DIY) test consists of a transducer made of graphite leads modified with human angiotensin-converting enzyme 2 (ACE2) receptor and a plastic vial (Fig. 1 *A–C*). This testing device enables on-site SARS-CoV-2 detection within 6.5 min, faster than tests currently approved by the Food and Drug Administration (FDA) (Fig. 1*D*). Each LEAD unit can be manufactured for a total cost of $1.50, including the vial ($0.30), graphite leads ($0.20), and all the modifiers required to ensure high sensitivity ($1.00), i.e., glutaraldehyde (GA), gold nanoparticles (AuNPs), cysteamine (cys), ACE2, and bovine serum albumin (BSA). The graphite working electrode (WE) was modified with AuNPs stabilized with cys to allow anchoring of ACE2 (16). BSA was used to block the remaining active sites on the electrode surface to avoid nonspecific interactions between the clinical sample and the biosensor. In summary, we describe a low-cost and rapid COVID-19 test that is easy to assemble and may enable population-wide high-frequency testing. Due to its reduced cost and DIY design, LEAD provides an opportunity to increase access to testing to underserved populations.

## Results

### Biosensor Design.

The electrochemical device was designed to explore the remarkable binding affinity between SARS-CoV-2 spike protein (SP) and human ACE2, its receptor in the human body (17, 18) (Fig. 1*A*). Thus, ACE2 was used as our recognition element to ensure sensitive and selective viral detection (19). The WE, where the (electro)chemical reaction/interaction takes place and is subsequently converted to a detectable analytical signal, was functionalized by the drop-casting method. The graphite WE was polished with a 2,000-grit sandpaper to remove impurities from the surface, and a contact area of 1.0-cm length by 0.7-mm diameter was obtained. Next, in order to generate a cross-linked polymer, the graphite pencil electrode (GPE) was immersed in a 25.0% (vol/vol) GA solution for 1 h at 37 °C. GA has been extensively used to modify electrodic surfaces as it introduces aldehyde functional groups that facilitate the covalent attachment of compounds containing amine terminal moieties (20, 21). Here, we leveraged GA to modify the GPE’s surface with AuNPs functionalized with cys (AuNPs-cys). First, we synthesized the AuNPs following protocols similar to those reported by our group previously (22). The AuNPs were functionalized with cys by their thiol groups. Next, the modified graphite substrate was kept under an AuNPs-cys solution at 37 °C for 75 min to allow the immobilization of AuNPs-cys by cross-linking between the aldehyde functional groups of GA and the amine functional groups of cys (20). Subsequently, we added a solution containing the preprepared reactive intermediary *N*-(3-dimethylaminopropyl)-*N*-ethylcarbodiimide hydrochloride (EDC) and *N*-hydroxysuccinimide (NHS) with ACE2, EDC-NHS-ACE2, to enable anchoring between the amine groups of AuNPs-cys and the EDC-NHS-ACE2, yielding ACE2-AuNPs-cys after 30 min at 37 °C. The electrodes were then incubated with BSA at 37 °C for 30 min in order to block the electrode’s remaining active sites after immobilization of ACE2 (Fig. 1*B*). BSA is a functionally inert protein with a high density of superficial lysine residues that is commonly used for biosensor development (23). Next, we exposed the sensor to samples containing SP or SARS-CoV-2 and changes in the peak current (ip) of a redox probe ([Fe(CN)_6_]^−3/−4^) enabled diagnosis of healthy samples versus those that were infected with SARS-CoV-2 (Fig. 1*C*).

### Characterization Assays.

**Fig. 2.**
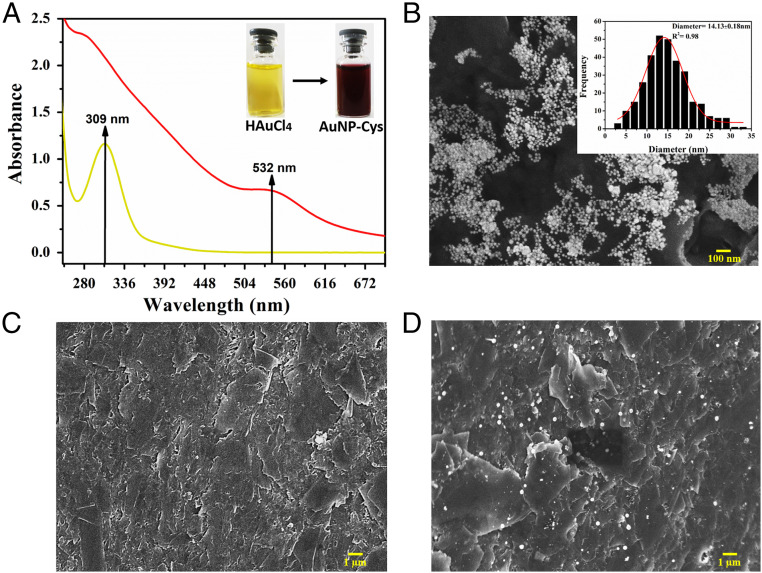
AuNPs-cys and GPE characterization studies. (*A*) UV-vis spectrum obtained for HAuCl_4_ solution (yellow color with a maximum absorbance band at 309 nm) and AuNPs-cys formation (wine color and absorbance band at 532 nm). (*B*) SEM image of AuNPs-cys dispersion with a histogram inset. The spherical AuNPs-cys present a mean diameter of 14.13 ± 0.18 nm. (*C*) SEM image of the bare GPE electrode polished showing a flat surface containing carbon sheets. (*D*) SEM image of the GPE electrode modified with GA and AuNPs-cys, which present a high AuNPs-cys distribution throughout the electrode, thus facilitating ACE2 immobilization and SARS-CoV-2 SP detection.

Experiments were then performed to characterize the biosensor. Fig. 2*A* presents an ultraviolet-visible (UV-vis) spectrum obtained from a HAuCl_4_ solution with a maximum absorption band at 309 nm. After AuNP-cys formation, a wine color was obtained displaying a UV-vis absorption band at 532 nm. Successful formation of the spherical AuNP-cys solution was confirmed by scanning electron microscopy (SEM) images (Fig. 2*B*), presenting a mean diameter of 14.13 ± 0.18 nm. The bare GPE presented a flat surface containing stacked carbon sheets (Fig. 2*C*). The AuNP-cys appeared well-distributed within the GPE surface (Fig. 2*D*) after the optimized functionalization process, facilitating the subsequent ACE2 immobilization onto the surface of the electrode.

### Electrochemical Characterization of the Biosensor.

**Fig. 3.**
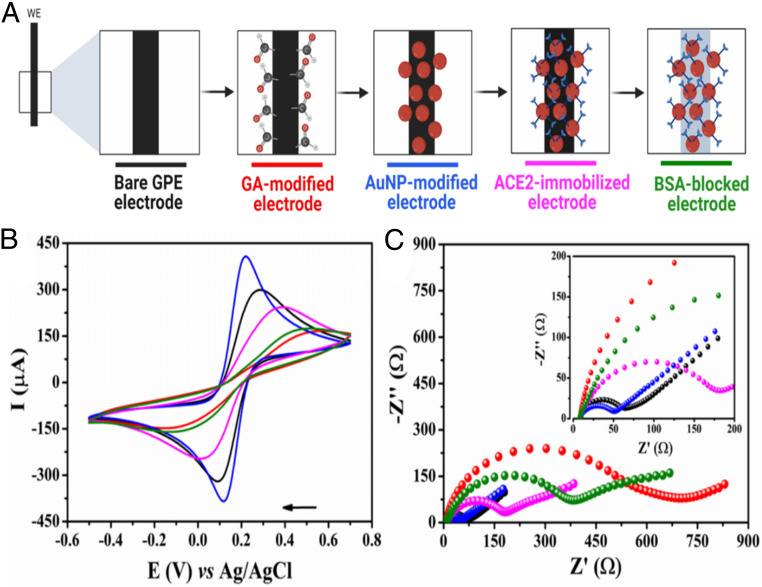
Functionalization steps and electrochemical characterization of LEAD. (*A*) Schematic representation of stepwise functionalization steps of LEAD. (*B*) CVs recorded for each modification step of the GPE surface in a solution of 5.0 mmol⋅L^−1^ [Fe(CN)_6_]^−3/−4^ containing 0.1 mol⋅L^−1^ KCl as the supporting electrolyte at a scan rate of 50 mV⋅s^−1^. (*C*) Nyquist plots were obtained using the same conditions as in *A*. (*Inset*) A zoomed view of the plots in high-frequency regions. The following experimental conditions were used for these experiments: frequency range from 1 × 10^5^ Hz to 0.1 Hz and 10-mV amplitude. All measurements were performed at room temperature.

The electrochemical behavior of each functionalization step (Fig. 3*A*) was analyzed by cyclic voltammetry (CV) and electrochemical impedance spectroscopy (EIS). CV (Fig. 3*B*) and Nyquist (Fig. 3*C*) plots revealed that the bare GPE electrode (black line) possessed a resistance to charge transfer (R_CT_) of 57.8 ±2.5 Ω, indicating a small resistance toward redox conversion and a high electron transfer on the electrode surface. This result was in agreement with the high ip of 303.3 ±13.6 µA shown for the same electrode by CV plot (black line). Next, we modified the WE with GA (red line), leading to an increased R_CT_ to 383.1 ± 8.3 Ω and decreased ip to 165.8 ± 6.1 µA. These data indicate that GA acts as an electrical insulator hindering the electron transfer at the interface of the WE by preventing the redox probe from reaching the GPE surface (24, 25). AuNPs-cys were then anchored covalently to the surface of the GPE (blue line) through an amide bond between the amine group from the cys and the aldehyde groups from the glutaraldehyde (26). The ACE2 anchoring step was successfully confirmed by the presence of an amide band at 1,650 cm^−1^ in our Fourier transform infrared (FTIR) spectroscopy analysis (*SI Appendix*, Fig. S1) (26, 27). The functionalization of GPE with AuNPs-cys led to decreased values of R_CT_ (47.6 ± 1.3 Ω) and increased ip (410.5 ± 13.7 µA) compared to the previous functionalization step (Fig. 3*A*). The higher current and lower charge transfer resistance detected resulted from the high electrocatalytic and surface area presented by the AuNPs, which contributed to fast electron-transfer kinetics and an active nanostructured electrodic surface (28, 29), thus conferring very attractive features for sensor development. In addition, the –NH_3_^+^ functional groups present on the AuNPs-cys–modified GPE led to favorable electrostatic interactions of the anionic probe [Fe(CN)_6_]^3−/4−^, providing a preconcentration of the redox probe close to the electrode interface. This led to an improved electrochemical response, i.e., higher current peak (30). The synthesized AuNP-cys dispersion demonstrated adequate stability for up to 7 d when stored at 4 °C under a light-protected environment (*SI Appendix*, Fig. S2). This stability allowed AuNP-cys synthesis to be carried out weekly, facilitating the large-scale modification process of GPE.

The electrodic functionalization with AuNPs-cys was evaluated through the redox behavior of the adsorbed AuNPs by CVs recorded using 0.1 mol⋅L^−1^ H_2_SO_4_ after different periods of immersion in the AuNPs-cys suspension (*SI Appendix*, Fig. S3). Indeed, an anodic peak was observed at +880 mV and a cathodic peak at +522 mV, which correspond to the redox processes of Au (III) within the electrode surface (*SI Appendix*, Fig. S3*A*). We next optimized the period of electrodic exposition to AuNP-cys suspension to be 80 min (*SI Appendix*, Fig. S3*B*) based on the peak current of the anodic process derived from oxidation of the gold adsorbed on the GPE/GA surface. Subsequently, we immobilized the recognition element ACE2 onto the surface of the nanoparticles-functionalized GPE using EDC and NHS (magenta line). This led to increased R_CT_ values (182.6 ± 2.2 Ω) and decreased ip values (247.2 ± 4.1 µA) compared to the previous functionalization step (*SI Appendix*, Fig. S3), confirming the proper anchoring of our recognizing element (ACE2) to the electrode surface, which led to the hindrance of the faradaic processes of [Fe(CN)_6_]^3−/4−^ taking place at the modified GPE surface. As a final functionalization step, we immobilized BSA (green line) to block the remaining unmodified electrodic area to avoid nonspecific and undesired adsorption of other molecules (e.g., proteins and lipids). This step resulted in the highest R_CT_ values (985.7 ± 12.7 Ω) and lowest ip (177.4 ± 3.2 µA), suggesting a continued decline in the charge transfer kinetics after anchoring the AuNPs-cys due to the insertion of nonconductive materials (i.e., ACE2 and BSA).

### Analytical Performance of LEAD.

We used square wave voltammetry (SWV) for SARS-CoV-2 detection. This technique is highly sensitive, especially for detecting reversible redox species (31), such as potassium ferri- and ferrocyanide. Our electroanalytical method is based on the signal suppression induced by highly specific interactions between the SP and ACE2, i.e., an increase in the SP concentration leads to a concomitant decrease in the current signal of the redox probe [Fe(CN)_6_]^−3/−4^. This means that the specific interaction between SP and ACE2 partially blocked the probe’s access to the surface of the WE (32). To achieve enhanced detection of SARS-CoV-2, we optimized the following instrumental parameters: frequency, amplitude, and step potential. The highest peak current values for the redox probe were obtained using a frequency of 80.0 Hz, amplitude potential of 75.0 mV, and step potential of 8.0 mV (*SI Appendix*, Fig. S4).

**Fig. 4.**
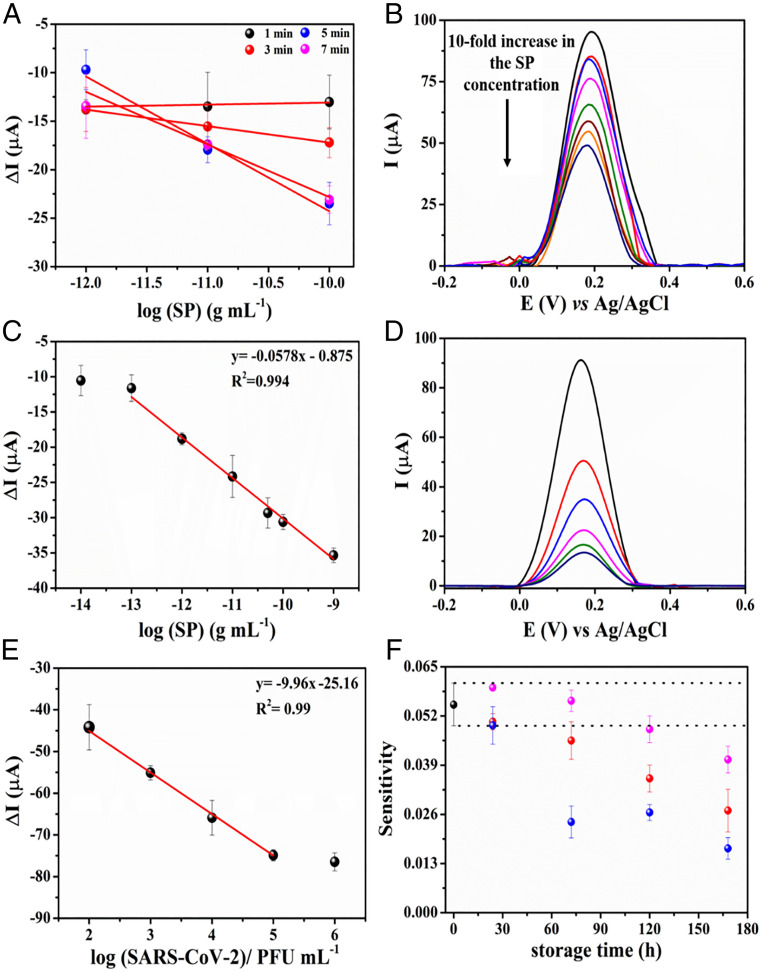
Kinetic study of the interaction between SARS-CoV-2 SP and LEAD. (*A*) Calibration curves built using SP at concentrations ranging from 1 × 10^−12^ g⋅mL^−1^ to 1 × 10^−10^ g⋅mL^−1^ and using different incubation times (ranging between 1 and 7 min). Increased analytical sensitivities were achieved after 5 min (6.94 × 10^−3^ ± 1.00 × 10^−3^) and 7 min (5.42 × 10^−3^ ± 6.30 × 10^−3^). Thus, 5 min was chosen as the optimal incubation time for LEAD. All measurements were recorded in triplicate, and each measure was done using a different sensor. (*B*) Baseline-corrected SWVs for the 5.0 mmol⋅L^−1^ [Fe(CN)_6_]^3−/4−^ redox probe after the electrode incubation with different concentrations of SP ranging from 1 × 10^−14^ to 1 × 10^−9^ g⋅mL^−1^. (*C*) Linear regression for the analytical curve constructed using the suppression current signal. Conditions: frequency, 80 Hz; amplitude, 70 mV, and step, 8 mV. All experiments were carried out in triplicate (*n* = 3), using 5.0 mmol⋅L^−1^ [Fe(CN)_6_]^3−/4−^ containing 0.1 mol⋅L^−1^ KCl as the supporting electrolyte after exposure of the electrode to 50 µL of standard SP solution for 5 min. (*D*) Baseline-corrected SWV plots for tittered-inactivated viral solutions at concentrations ranging from 10^2^ to 10^6^ PFU⋅mL^−1^ in VTM. (*E*) Linearized correlation between the ΔI values and concentration of inactivated virus in solution. The analytical curve was carried out in triplicate measurements using different LEAD devices. (*F*) Stability study under different storage conditions: 25 °C (black circles), −20 °C (red circles), and stored dry at 4 °C (blue circles) and stored at 4 °C in PBS medium (pH = 7.4) (magenta circles) over 7 d. Sensitivity values were obtained by analytical curves in the concentration range from 1 × 10^−12^ g⋅mL^−1^ to 1 × 10^−9^ g⋅mL^−1^ of SP. All experiments were carried out in triplicate (*n* = 3).

Next, we evaluated the optimal incubation time for detecting SARS-CoV-2 in clinical samples by evaluating the analytical sensitivity parameter obtained from dose–response curves at very low SP concentrations (Fig. 4*A*). The experiments were recorded in triplicate using increased concentrations of SP, from 1 × 10^−12^ to 1 × 10^−10^ g⋅mL^−1^. The results were expressed as $\Delta I=I-I_{0}$, where I corresponds to the current recorded for the redox probe ([Fe(CN)_6_]^3−/4−^) after incubating the sample on the electrode surface and $I_{0}$ corresponds to the current recorded for the redox probe before exposing the biosensor to the sample (Fig. 4*A*). Five minutes was determined to be the optimal incubation time due to the highest value of the angular coefficient of the dose–response curves, demonstrating fast binding kinetics between the SARS-CoV-2 SP and the immobilized ACE2 on the electrode surface.

We also obtained an analytical curve for different concentrations of SP in 0.1 mol⋅L^−1^ phosphate-buffered saline (PBS) (pH = 7.4) under optimized experimental conditions (Fig. 4*B*). Note that the SWV response for the redox probe [Fe(CN)_6_]^3−/4−^ decreased with increased concentration of SP due to suppression of the analytical signal (ip) induced by the highly specific interaction between the SP and the ACE2-modified GPE (17, 33). Binding of SP to the biosensor surface partially blocked the electroactive sites of LEAD, leading to current suppression and yielding a positive result indicative of the presence of SARS-CoV-2 SP.

The SWV signals (*n* = 3 measurements using different biosensors) obtained at each concentration were plotted as a logarithmic function of the SP concentration (Fig. 4*C*). The analytical curve (Fig. 4*C*) was calculated at concentrations ranging from 1 × 10^−14^ g⋅mL^−1^ to 1 × 10^−9^ g⋅mL^−1^ of SP and displayed a linear behavior at the concentration range between 1 × 10^−13^ g⋅mL^−1^ and 1 × 10^−9^ g⋅mL^−1^ SP, resulting in an analytical sensitivity value of 0.0575 ± 0.0020 µA⋅g^−1^⋅mL^−1^ and a high linear correlation (R^2^) of 0.994.

The limit of detection (LOD) and limit of quantification (LOQ) of LEAD were calculated according to the four-parameter logistic (4PL) curve (*SI Appendix*, Fig. S5), using Eqs. 1 and **2** (34). This method is commonly used for assays that determine biological binding interactions and reflect the underlying binding kinetics (35–37). Thus, the LOD and LOQ values of LEAD were 229 fg⋅mL^−1^ and 0.91 pg⋅mL^−1^, respectively. Therefore, our device enabled the rapid detection of SP at very low concentrations (less than picograms per milliliter), providing high sensitivity (Fig. 4*C*) and a low LOD using highly accessible materials, such as pencil graphite and a plastic vial. In *SI Appendix*, Table S1 we show a side-by-side comparison of the performance of LEAD and other electrochemical detection methods described for SARS-CoV-2 diagnosis. Note that LEAD enables high detectability (LOD = 229 fg⋅mL^−1^), rapid testing time (6.5 min), and a very low production cost ($1.50 per test). The testing time was recorded to be 6.5 min considering the sample incubation period (5 min), the time required to record two SWVs (before and after sample incubation, 1 min), and the washing step with PBS after incubating the sample (30 s).

To assess the diagnostic capability of LEAD we calibrated our biosensor using tittered solutions of inactivated SARS-CoV-2 ranging from 10^2^ to 10^6^ plaque-forming units (PFU)⋅mL^−1^ (Fig. 4 *E* and *F*). LEAD exhibited high sensitivity, presenting an LOD of 2.07 PFU⋅mL^−1^, corresponding to the order of 10 RNA copies per microliter, which is similar to RT-qPCR sensitivity (38, 39).

$$L_{C}=\;{µ}_{\text{blank}}+t\left(1-\alpha ,\;\text{n}-1\right)\sigma_{\text{blank}},$$ [1]

where $L_{C}$ is a value of blank limit, ${\;µ}_{\text{blank}}$ is the mean of signal intensities for n blank (negative control) replicates, $\sigma_{\text{blank}}$ is the SD of blank replicates, andt(1−α, n−1) is the 1−α percentile of the t-distribution given n−1 degrees of freedom, α = β = 0.05 significance levels.

$$\;L_{d}=\;L_{C}+t(1-\beta ,\;\text{m}\left(\text{n}-1\right)\sigma_{\text{test}},$$ [2]

where $L_{d}$ is the LOD in the signal domain, $\sigma_{\text{test}}$ is the pooled SD of n test replicates, and t(1−β, m(n−1) is the 1−β percentile of the t-distribution given m(n−1) degrees of freedom. Again, we set α = β = 0.05, but these significance levels can be selected depending on the needs of a given study.

Our electrochemical biosensor was applied for SARS-CoV-2 detection in clinical samples containing a wide range of viral loads. The threshold cycle (Ct) of the RT-PCR data for all clinical samples analyzed ranged from 21.5 to 34.3 Ct. It is important to highlight that our results (current suppression – ΔI) presented a high linear correlation (*R*^2^ = 0.954) with Ct values ranging from 22.3 to 34.3 (*SI Appendix*, Fig. S6).

### Batch-to-Batch Reproducibility and Stability Assays.

We performed reproducibility assays of our device to ensure that different test batches performed similarly. To verify the reproducibility of the manufacturing and functionalization processes of LEAD we recorded SWVs in the presence of 5 mmol⋅L^−1^ [Fe(CN)_6_]^−3/−4^ after incubating the sensor with 1 × 10^−12^ g⋅mL^−1^ of SP prepared in 0.1 mol⋅L^−1^ PBS (pH = 7.4). A relative SD of 4.31% was obtained for the analytical signal (current suppression of the redox probe) using six sensors (*n* = 6) from different batches, indicating excellent reproducibility (*SI Appendix*, Fig. S7).

Next, we evaluated the stability of LEAD under different temperature and storage conditions (25 °C, stored dry at 4 °C, stored at 4 °C in PBS, and at −20 °C) over 7 d. Analytical curves were generated in 0.1 mol⋅L^−1^ PBS (pH = 7.4) at a concentration ranging from 1 × 10^−12^ g⋅mL^−1^ to 1 × 10^−9^ g⋅mL^−1^ of SP (Fig. 4*D*). Sensors stored under dry conditions at 4 °C (without PBS) were stable for 24 h. However, after 72 h the stability decreased to 50% of the initial sensitivity (Fig. 4*F*). Interestingly, electrodes stored at 4 °C in PBS solution were stable for 120 h, and the mean sensitivity of the device decreased by only 23% after 6 d compared to the initial performance of LEAD (Fig. 4*F*). These results demonstrate that the immobilized ACE2 maintained its activity over prolonged periods of time (up to 5 d) when stored in a refrigerated aqueous solution.

### Cross-Reactivity Experiments.

**Fig. 5.**
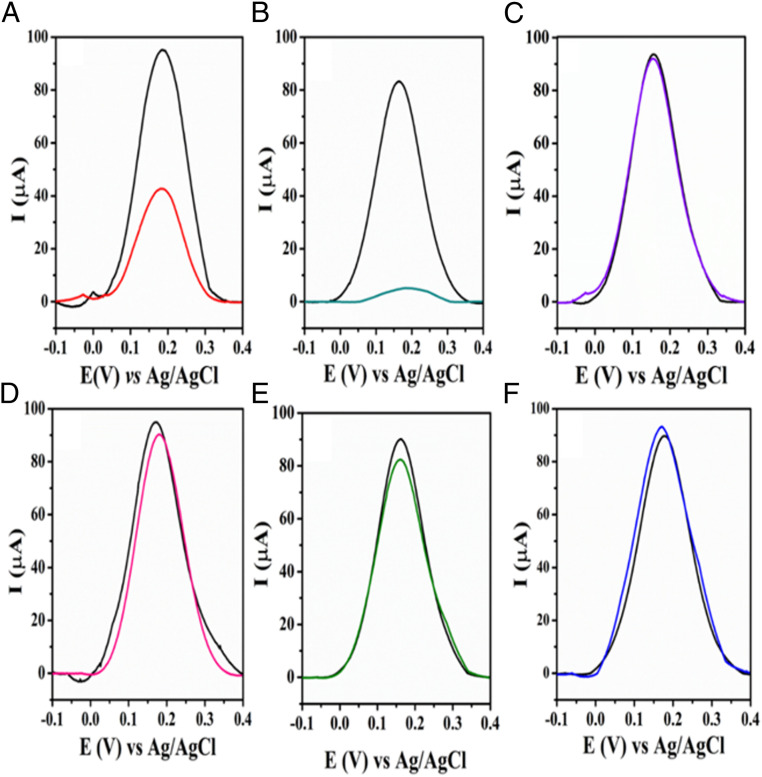
Cross-reactivity studies of LEAD using other coronaviruses and noncoronavirus strains. Baseline-corrected SWVs recorded at optimized experimental conditions before (black lines) and after incubation of the electrode with (*A*) SARS-CoV-2, (*B*) SARS-CoV-2 UK variant B, and possible interfering viruses such as (*C*) H_1_N_1_-A/California/2009, (*D*) Influenza-B/Colorado, (*E*) herpes simplex virus-2, and (*F*) MHV. The following conditions were used for all experiment: frequency of 80 Hz, amplitude of 70 mV, and step potential 8 mV. All experiments were performed in 5.0 mmol⋅L^−1^ [Fe(CN)_6_]^−3/−4^ in 0.1 mol⋅L^−1^ KCl. The specificity studies were carried out using the following viral strains: MHV at 10^8^ PFU⋅mL^−1^ (coronavirus); H_1_N_1_, A/California/2009; Influenza B, B/Colorado; HSV2, and herpes simplex virus-2 (all at 10^5^ PFU⋅mL^−1^). The analysis of the contagious SARS-CoV-2 B.1.1.7 UK variant showed that the variant presented higher interaction between SP and ACE2 compared to SARS-CoV-2. Each virus was incubated in a final volume of 50 µL for 5 min. All viruses were in stored VTM and experiments were performed at room temperature.

Cross-reactivity studies using other viruses were carried out to investigate the specificity of our biosensor toward SARS-CoV-2 and rule out potential off-target reactivity. Using the same experimental conditions as for SARS-CoV-2 (Fig. 5), we tested four other viral strains: H_1_N_1_ (A/California/2009), Influenza-B/Colorado, herpes simplex virus-2, and murine hepatitis virus (MHV). No cross-reactivity was detected against any of these viruses as indicated by the response to all strains, which presented a current suppression (ΔI) lower than the cutoff value of 10 µA obtained by SWV for the lowest SP concentration detected (Fig. 4*C*). These results further highlight the translatability of our sensor toward COVID-19. Interestingly, LEAD displayed a higher affinity to the highly infectious SARS-CoV-2 UK variant 1.1.7.B (Fig. 5*B*) than to wild-type SARS-CoV-2 (Fig. 5*A*), which is in agreement with recent studies demonstrating that mutations in the receptor-binding domain of the spike glycoprotein variant led to enhanced binding affinity toward ACE2 (40, 41).

### Detection of SARS-CoV-2 in Clinical Samples.

To evaluate the diagnostic efficacy of LEAD, we tested 103 NP/OP (Table 1) and 10 saliva samples (*SI Appendix*, Tables S2 and S3) obtained from inpatients from the Hospital of the University of Pennsylvania after heat inactivation. All samples were confirmed as COVID-19 positives and COVID-19 negatives by RT-PCR. Table 1 shows that out of the 103 NP/OP samples tested (53 COVID-19–positive and 50 COVID-19–negative samples) our device accurately detected 90 (88.7% sensitivity, 86.0% specificity, and 87.4% accuracy). To evaluate the diagnostic efficacy of our device in a more complex biological environment, we tested 10 saliva samples (3 positive and 7 negative samples) as a proof of principle. LEAD presented higher accuracy, sensitivity, and specificity (100.0%) for saliva samples than for NP/OP swabs, although the sample size was substantially different between the two groups.

**Table 1.** Detection of SARS-CoV-2 in OP/NP and saliva samples by LEAD

	RT-qPCR			Sensitivity	Specificity	Accuracy
	Positive	Negative	Total			
NP swabs						
Positive	47	7	54	47/53 (88.7%)		90/103 (87.4%)
Negative	6	43	49		43/50 (86.0%)	
Saliva						
Positive	3	—	3	3/3 (100.0%)		10/10 (100.0%)
Negative	—	7	7		7/7 (100.0%)	

Analytical features of LEAD obtained using clinical samples. The sensitivity, specificity, and accuracy of LEAD for NP/OP and saliva samples were measured. Positive and negative values for the clinical samples were obtained by RT-qPCR.

## Discussion

We present a simple, inexpensive, and portable electrochemical biosensor that enables diagnosis of COVID-19 within 6.5 min using 50 µL of sample and highly accessible and commercially available materials (i.e., graphite pencil leads and a plastic vial), yielding a test that costs $1.50. The WE can be functionalized in less than 3 h and remains stable for over 5 d when stored in a PBS solution at 4 °C. LEAD displayed excellent sensitivity for detecting SARS-CoV-2 SP (LODs of 229 fg⋅mL^−1^ and 2.07 PFU⋅mL^−1^ for free SP in PBS medium and inactivated virus in virus transportation medium [VTM] medium, respectively) and did not cross-react with other relevant viruses.

The robustness and accuracy of LEAD were successfully evaluated by analyzing 113 clinical samples (both OP/NP and saliva) including the highly infectious SARS-CoV-2 UK variant 1.1.7.B, indicating that our method does not require further adaptations to accurately diagnose the SARS-CoV-2 UK variant B.1.1.7. However, additional variants would have to be tested to assess the broad applicability of LEAD, particularly when considering emerging mutants. Based on its low cost, selectivity, and accuracy features, LEAD constitutes a potential candidate for high-frequency testing at the point of care and may enable population surveillance and pandemic outbreak control since the modification of the electrodes can be performed in batch and the method can be automated to facilitate mass production. Collectively, our technology may help limit asymptomatic spread through high-frequent testing, i.e., multiple times per week (22), since it is sufficiently inexpensive (U$1.50), rapid (results within 6.5 min), and easy to assemble and use. Finally, LEAD can be applied beyond COVID-19 for the detection of other emerging pathogens (e.g., other viruses, bacteria, fungi, etc.) as long as the infectious agent and its receptor are available.

## Materials and Methods

### Materials.

All reagents used in this work were of analytical grade. The deionized water (resistivity ≥18 MΩ cm at 25 °C) was obtained from a Milli-Q Advantage-0.10 purification system (Millipore). ACE2 Fc Chimera, Human was obtained from GenScript. Spike protein was kindly donated by Scott Hensley, University of Pennsylvania, Philadelphia. EDC and NHS with a degree of purity ≥98%, gold(III) chloride trihydrate (HAuCl_4_0.3H_2_O) (99.99%), sodium borohydride (NaBH_4_) with ≥98% purity, cysteamine hydrochloride (cys) with 98% purity, phosphate buffer saline solution, pH = 7.4, and glutaraldehyde (25%, vol/vol) were purchased from Sigma-Aldrich. Graphite pencils 0.7-mm and 0.9-mm diameter under the trade name of Pentel were purchased in a local store in Philadelphia. Ag/AgCl conductive ink was acquired from Creative Materials.

Electrochemical measurements were carried out using a MULTI AUTOLAB M101 potentiostat with six channels, controlled by the NOVA 2.1 software. Cryogenic vials were used as the electrochemical cell (2.0-mL volume), each containing three electrodes. Graphite pencils (0.9 mm) were used as reference and counterelectrodes, and graphite pencils (0.7 mm) were used as the WE. The Ag/AgCl ink was painted over one of the graphites to create the reference electrode.

### Characterization Studies.

Electrochemical techniques, such as CV, were carried out in a potential window from 0.7 to −0.3 V with a scan rate of 50 mV⋅s^−1^. EIS was conducted in the frequency range from 1 × 10^5^ Hz to 0.1 Hz using an amplitude of 10 mV and under open circuit potential. Both electrochemical techniques were used to characterize the electrochemical behavior of electrodes in each modification step. In SWV studies, potentials were scanned from −0.4 to 1.0 V, corresponding to a frequency of 80 Hz, amplitude of 75 mV, and step low of 8 mV. All electrochemical characterizations were performed in 0.1 mol⋅L^−1^ KCl solution containing 5.0 mmol⋅L^−1^ of the mixture [Fe(CN)_6_]^3−/4−^ solution. All electrochemical experiments were carried out at room temperature (25 ± 3 °C).

Morphological characterizations of the synthesized AuNP-cys dispersion and GPE before and after superficial functionalization with AuNP-cys/ACE2/BSA were performed using SEM images acquired using a JEOL 7500F HRSEM from the Singh Center for Nanotechnology (University of Pennsylvania). SEM images were recorded with 15,000 to 150,000× magnifications, acceleration voltage of 5 kV, and using SEI mode. The spectrophotometry analysis was performed using a PerkinElmer Multimode Plate Reader spectrophotometer (model EnVision). The absorbance of AuNP-cys dispersion was measured at 532 nm (at room temperature) over 7 d to evaluate its stability. FTIR spectra were obtained for GPE/AuNP-cys and GPE/AuNP-cys/ACE2 samples in a PerkinElmer Spectrum 2 equipped with a Diamond UATR 2 detector ranging from 500 cm^−1^ to 4,500 cm^−1^ and 32 scans were performed for each measurement.

### Synthesis of AuNPs.

The AuNPs-cys were prepared according to methodologies described in the literature (42, 43). First, 100 µL of cys (213.0 mmol⋅L^−1^) was dropped into 1.5 mmol⋅L^−1^ HAuCl_4_ in a final volume of 10.0 mL, under vigorous stirring for 20 min at room temperature. Subsequently, 10.0 µL of NaBH_4_ (10.0 mmol⋅L^−1^) was added and kept under stirring for 20 min in a light-protected environment at room temperature. The resulting yellow color changed to wine red as a consequence of the formation of AuNPs. Finally, the solution was stored at 4 °C in a refrigerator for up to 7 d, conditions at which it presents high stability when stored in the absence of light (*SI Appendix*, Fig. S1) (42, 44).

The cys presents an amine group (−NH_3_^+^) and a mercaptan group (−SH) in its extremities, the latter of which favorably binds onto the AuNPs surface through an Au–S bond. Thus, cys serves to provide greater stability to the AuNPs due to the electrostatic repulsion process among the amine groups, which generates free positive charges (26, 43, 45).

### Modification of Graphite Lead Electrodes.

The WE was polished with 2,000-grit sandpaper, and a contact area of 1.0-cm length by 0.7-mm diameter was obtained. Next, the GPE was kept immersed in a 25.0% (vol/vol) glutaraldehyde solution for 1.0 h as a first modification step. This process allowed the graphite surface to be functionalized with aldehyde groups. Then, the modified graphite substrate was kept in a AuNPs-cys solution with protonated amine groups from cys at pH 7.4, which enabled covalent anchoring of the AuNPs to the substrate by the formation of an amide bond (26). The AuNPs-cys presented a maximum adsorption time of 75 min and, after 100 min, the AuNPs-cys presented low adsorption to the substrate, and its color gradually faded (*SI Appendix*, Fig. S2).

Subsequently, the solution containing 50 mmol⋅L^−1^ EDC, 25 mmol⋅L^−1^ NHS, and 10 µg⋅mL^−1^ of ACE2 diluted in 0.1 mol⋅L^−1^ PBS (pH = 7.4) was incubated on the substrate containing GPE/GA/AuNPs-cys. After 30 min, the ACE2 immobilized onto the substrate reached the anchoring stability and provided a highly sensitive SWV response. In the presence of EDC and NHS, the carboxyl group of ACE2 bound to the primary amine of cys through a covalent bond. The reaction between carboxyl groups and EDC–NHS resulted in the formation of a stable ester, which undergoes nucleophilic substitution with the amine groups present on the substrate surface (AuNPs-Cys), resulting in the formation of an amide bond between the modified GPE/GA/AuNPs-cys surface and ACE2. In the final step, nonspecific sites present within the electrode surface were blocked by incubation in a 1% (mass/vol) BSA solution for 30 min. Fig. 1 illustrates the simplified process for modifying LEAD (32, 46).

### COVID-19 Sensing Using LEAD.

For diagnosing SARS-CoV-2, a volume of 50 μL of VTM or a 0.1 mol⋅L^−1^ PBS sample containing SP was applied to the WE using a plastic vial and incubated for 5 min. After the incubation period, the WE was gently washed with 0.1 mol⋅L^−1^ phosphate buffer solution (pH = 7.4) to remove unbound virus or sample. Next, the electrodes were stored in a 2.0-mL cryogenic vial for subsequent electrochemical detection. One milliliter redox probe solution (5.0 mmol⋅L^−1^ [Fe(CN)_6_]^3−/4−^ in 0.1 mol⋅L^−1^ KCl) was used for the voltammetric measurements and to detect the current suppression due to binding of SARS-CoV-2 SP to the biosensor. Subsequently, the electrochemical response was monitored using the SWV technique. Notably, this procedure was applied to other studies such as cross-reactivity, reproducibility, incubation time, and SARS-CoV-2 detection in clinical samples. The total diagnostic time was calculated to be 6.5 min considering the sample incubation period (5 min), the time required to record two SWVs (before and after sample incubation, 1 min), and the washing step after sample incubation (30 s).

### Reproducibility, Stability, and Cross-Reactivity Studies.

The reproducibility study was performed by recording the analytical signal (current suppression of the redox probe) obtained for six different sensors (from different batches) after incubation with 1 pg⋅mL^−1^ SP solution. The stability of LEAD was evaluated by the analytical sensitivity value extracted from analytical curves in a concentration range from 1 × 10^−12^ to 1 × 10^−10^ g⋅mL^−1^ of SP in different conditions 25 °C, −20 °C, stored dry at 4 °C, and stored at 4 °C in PBS medium (pH = 7.4) over 7 d. All modified electrodes were stored inside capped plastic vials during the stability study.

The specificity studies were carried out using the following different viruses: MHV at 10^8^ PFU⋅mL^−1^ (coronavirus); H_1_N_1_, A/California/2009; Influenza B, B/Colorado; and HSV2, herpes simplex virus-2 (all at 10^5^ PFU⋅mL^−1^). The highly contagious SARS-CoV-2 B.1.1.7 UK variant was used to assess the capability of LEAD to detect SARS-CoV-2 variants.

### Clinical Sample Analyses.

The clinical performance of LEAD was assessed using clinical samples acquired from the Hospital of the University of Pennsylvania and deidentified prior to use. We set a current suppression (ΔI) cutoff value higher than 10 µA for diagnostic purposes in accordance with the analytical response obtained for the lowest concentration of SP detected (10^−14^ g⋅mL^−1^) in the dose–response curve (Fig. 4*C*), i.e., samples that exhibited ΔI >10 µA were deemed positive for SARS-CoV-2 infection. One hundred and three NP/OP swab samples in VTM were obtained from patients and heat-inactivated (50 negatives and 53 positives). We also used 10 saliva samples (3 positives and 7 negatives). All samples were analyzed and the results obtained were compared to those from RT-PCR (*SI Appendix*, Tables S2 and S3). The Ct values obtained by RT-PCR for the clinical samples ranged from 21.5 to 34.3.

## Supplementary Material

Supplementary File

## Data Availability

All study data are included in the article and/or *SI Appendix*.
