# Supplementary material for: Minute-scale detection of SARS-CoV-2 using a low-cost biosensor composed of pencil graphite electrodes
Source: Proc Natl Acad Sci U S A. 2021 Jul 9;118(30):e2106724118. doi: 10.1073/pnas.2106724118 (PMC8325344; doi:10.1073/pnas.2106724118)
Supplement: Supplementary File [file pnas.2106724118.sapp.pdf]

PNAS
www.pnas.org

Supplementary Information for
Minute-scale detection of SARS-CoV-2 using a low-cost biosensor composed of pencil graphite electrodes

Lucas F. Lima[1-4], André L. Ferreira[1-4], Marcelo D. T. Torres[1-3], William R. de Araujo[4]*, Cesar de la Fuente-Nunez[1-3]*.

*Cesar de la Fuente-Nunez and William R. de Araujo.
Email: cfuente@upenn.edu; wra@unicamp.br

This PDF file includes:

Figures S1 to S7
Tables S1 to S3
SI References

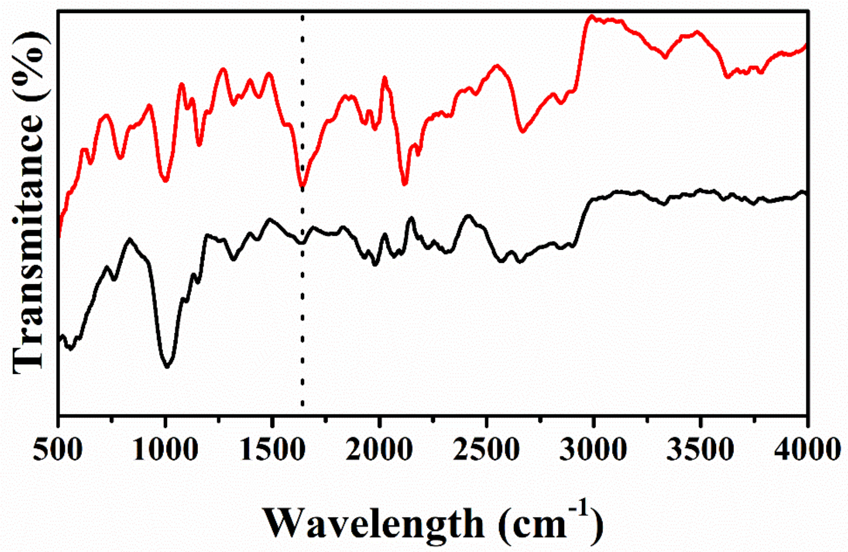

Figure S1. FTIR spectra recorded for the graphite modified with AuNP-cys after the functionalization step with EDC-NHS-ACE2 to confirm the covalent attachment of the enzyme due to amide functional group formation. GPE/GA/AuNP-cys (black line) and GPE/GA/AuNP-cys/ACE2 (red line).

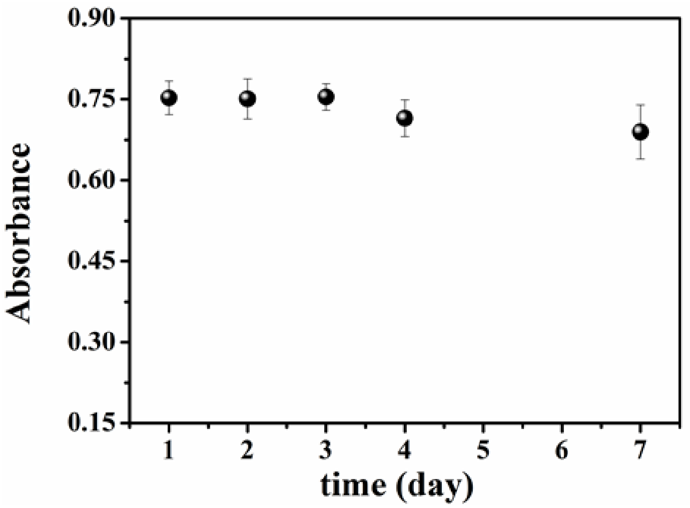

Figure S2. Stability of AuNP-cys suspension over seven days of storage at 4 ºC. AuNP-cys presented high stability under the conditions evaluated for up to seven days (stored at 4 ºC in a light-protected environment). All measurements were recorded in quintuplicate (n = 5) at room temperature using a spectrophotometer with a wavelength of 532 nm.

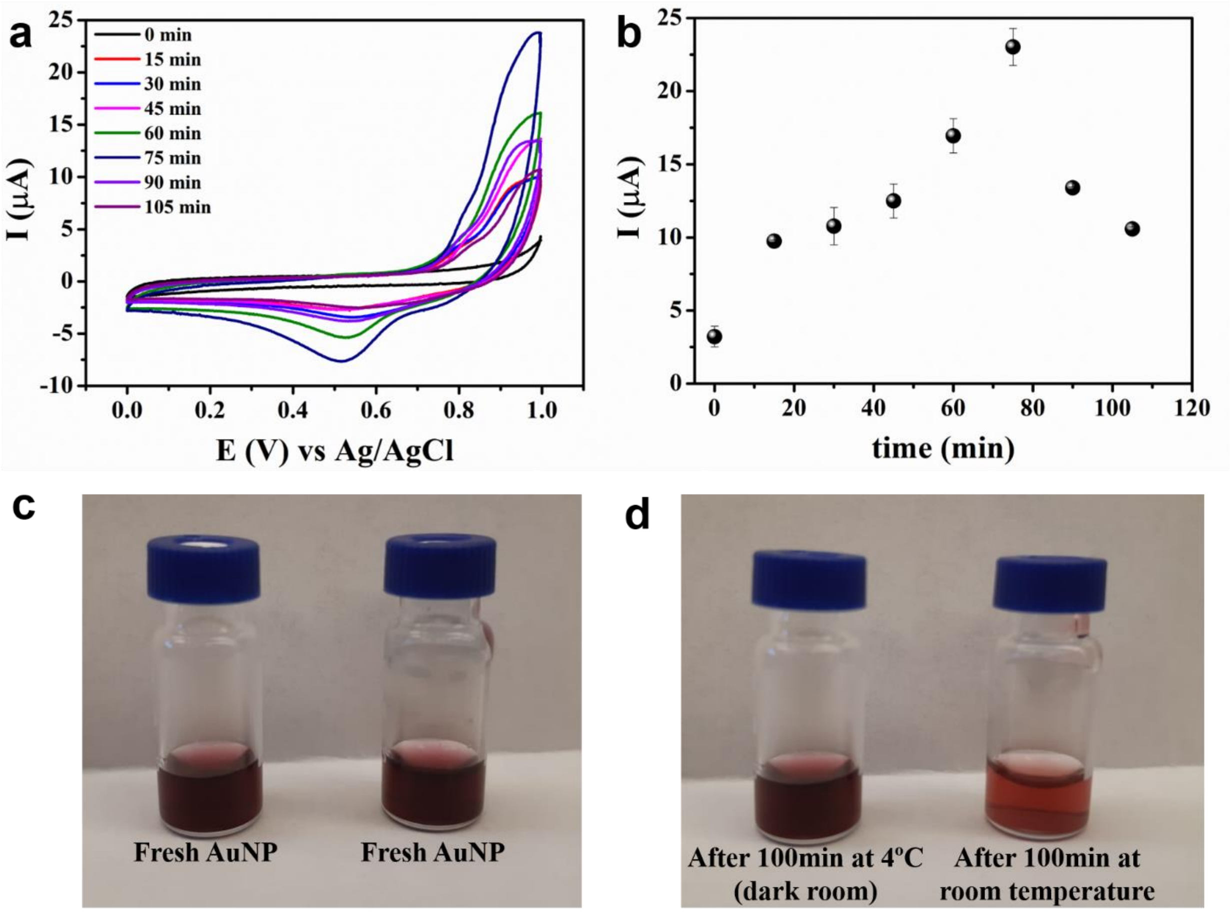

Figure S3. Functionalization study of AuNP-cys on glutaraldehyde-modified GPE for different periods of exposition to nanoparticle suspension. (a) CV plots in 0.1 mol L^{-1} H_2SO_4 with a scan rate of 50 mV s^{-1} in a potential window from 0.0 to 1.0 V with an anodic peak at +880 mV and a cathodic peak at +522 mV, which are attributed to the redox processes of Au (III) in electrode surface. AuNPs are oxidized at +880 mV on the anodic scan and converted back at +522 mV on the reverse cathodic scan (1). **(b)** peak current of anodic process related to oxidation of the gold adsorbed on the GPE/GA surface along the time of electrodic exposition to AuNP-cys suspension; **(c)** Plasmonic effect observed for fresh AuNP-cys before light exposure; **(d)** Plasmonic effect observed after 100 min for AuNP-cys stored in the refrigerator at 4 ºC and at room temperature. Note that the color of AuNP-cys faded after 100 min of exposure to light and at room temperature due to nanoparticle degradation. These results explain the decrease in the accumulation of this nanomaterial on the electrodic surface for long periods of exposure (> 80 min).

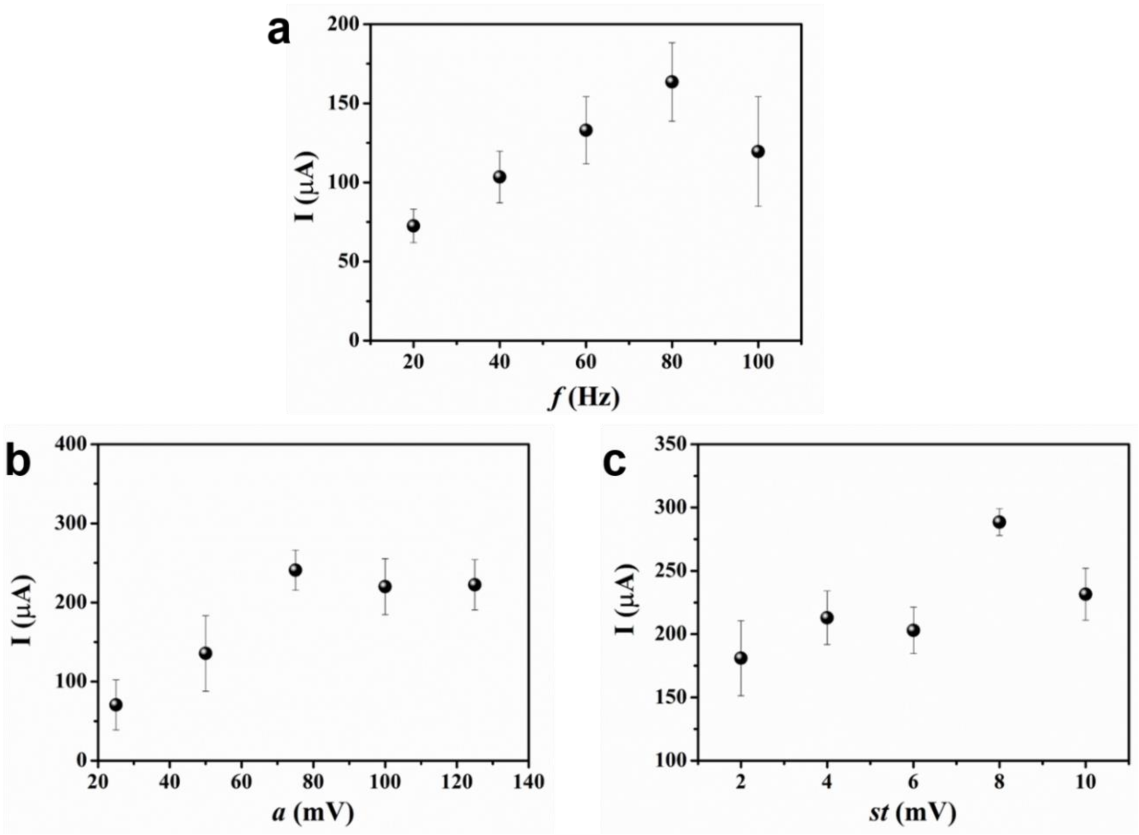

Figure S4. Optimization study of SWV parameters for electrodes incubated with 1 ng mL^{-1} of spike protein. (a) Optimization of the frequency using the SWV method, **(b)** optimization of the modulation amplitude, and **(c)** optimization of the applied step potential. All measurements were recorded in triplicate (n = 3). The optimal conditions for SWV response were obtained for the frequency of 80 Hz, amplitude of 75 mV, and step of 8 mV.

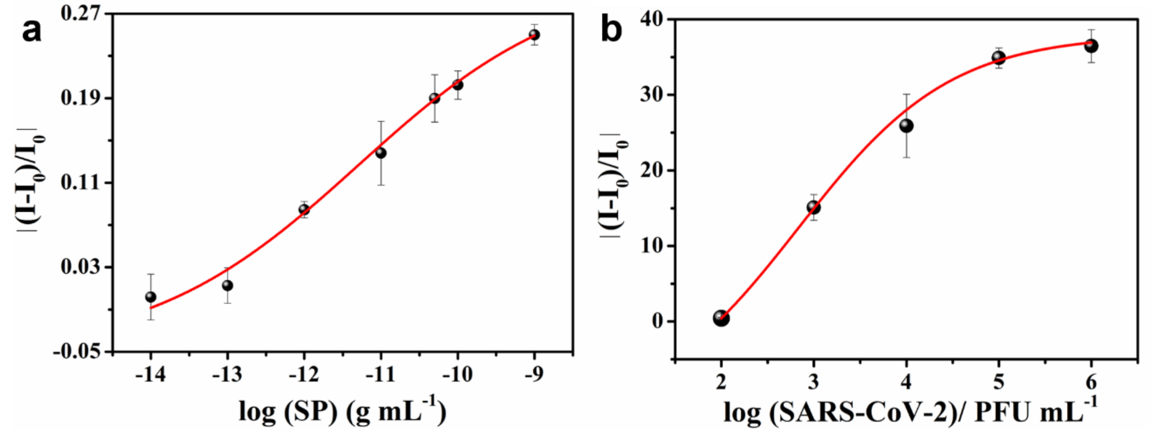

Figure S5. Normalized analytical curves [$(I-I_0)/I_0$] plotted to calculate the limit of detection using the four-parameter logistic 4PL method. (a) Analytical curve constructed using the normalized suppression current signal. The conditions used were the

following: frequency at 80 Hz; amplitude at 70 mV and step at 8 mV. All experiments were carried out in triplicate (n = 3), using 5.0 mmol L^{-1} $[Fe(CN)_6]^{3-/4-}$ containing 0.1 mol L^{-1} KCl as the supporting electrolyte after the electrode was exposed during 5 min to 50 µL of standard SP solution. **(b)** Analytical curve constructed using the normalized values of current in the function of the logarithmic concentration of inactivated virus in solution. The analytical curve was carried out in triplicate measurements using different LEAD sensors.

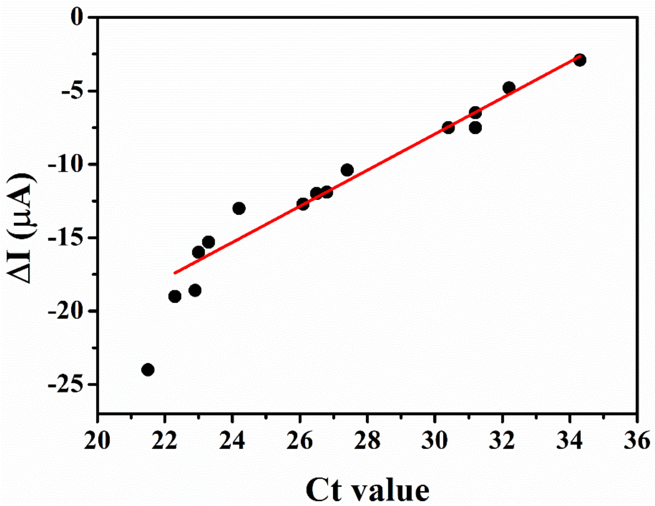

Figure S6: Correlation between the electrochemical results obtained with LEAD (current suppression – ΔI) and RT-PCR Ct values from clinical samples. The Ct values of the 16 representative clinical samples analyzed ranged from 21.5 to 34.3 and a linear correlation was obtained in the Ct range from 22.3 to 34.3, with an $R^2 = 0.954$.

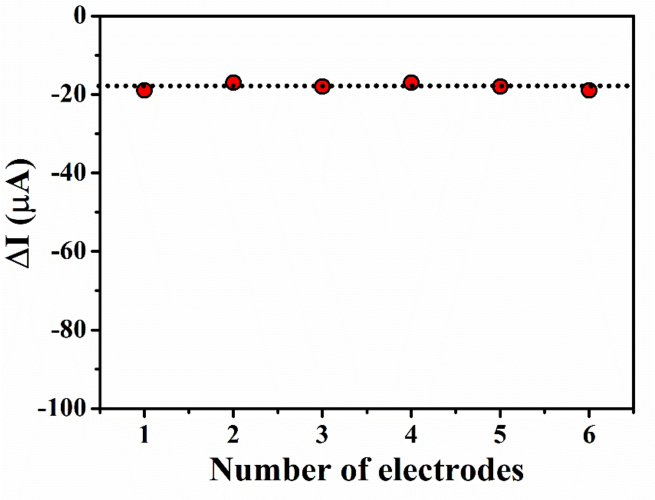

Figure S7: Reproducibility study. Plot showing the analytical response (current suppression of the redox probe) obtained for 6 differents LEAD when incubated with $1x10^{-12}$ g mL^{-1} of SP over 5 minutes. A relative standard deviation (RSD) of 4.31% was obtained, indicating an excellent reproducibility in the fabrication method.

Table S1. Analytical feature comparison of different electrochemical sensors developed for SARS-CoV-2 detection.

Sensor	LOD	Target	Electrochemical technique	Linear range	Time (min)	Reference
**LEAD**	1.96 $x10^{-13}$ g mL^{-1}	SP	SWV	$1x10^{-14}$ to $1x10^{-9}$ g mL^{-1}	6.5	This work
**Multiplex RCA**	1 copy μL^{-1}	N and S gene	DPV	1 to $1x10^{9}$ copies μL^{-1}	31.0	(2)
**Paper/GO/SARS-CoV-2 antibody**	9.6 $x10^{-10}$ g mL^{-1}	SARS-CoV-2 IgG and IgM	SWV	$1x10^{-9}$ to $1x10^{-6}$ g mL^{-1}	46.0	(3)
**SPE/CB/ SARS-CoV-2 antibody**	19 $x10^{-9}$ g mL^{-1}	N and S protein	DPV	$0.5x10^{-4}$ to $20x10^{-6}$ g mL^{-1}	31.0	(4)
**Au@SCX8-TB-RGO-LP- Target/ Au@Fe_3O_4**	200 copies mL^{-1}	RNA of SARS-CoV-2	DPV	$1x10^{-17}$ to 10^{-12} mol L^{-1}	180.0	(5)
**Cotton-tipped electrode/SARS-CoV-2 antibody**	8.0 x 10^{-13} g mL^{-1}	N protein	SWV	$1x10^{-12}$ to $1x10^{-6}$ g mL^{-1}	21.0	(6)
**MIP/ncovP**	1.5 $x10^{-14}$ g mL^{-1}	SARS-CoV-2 nucleoprotein	DPV	$2x10^{-15}$ to $1.11x10^{-13}$ g mL^{-1}	15.0	(7)
**RAPID**	2.18 $x10^{-15}$ g mL^{-1}	SP	EIS	$1x10^{-13}$ to $1x10^{-7}$ g mL^{-1}	4.0	(8)

**Graphene-based FET/ SARS-CoV-2 antibody**	1.0 x10^{-13} g mL^{-1}	SP	Chronoamperometry	1x10^{-13} to 1x10^{-10} g mL^{-1}	243.0	(9)

GO: graphene oxide; MIP: molecular imprinted polymers; ncovP: nucleoprotein P; DPV: differential pulse voltammetry; FET: field-effect transistor; RCA: rolling circle amplification; SPE: Screen-printed electrode; CB: carbon black; Au@SCX8-TB-RGO:p-sulfocalix[8]arene (SCX8) functionalized graphene (SCX8-RGO); targeting RNA of SARS-CoV-2; label probe (LP); Au@Fe_3O_4: magnetite gold nanoparticles. Time (min) is equal incubation time plus measure.

Table S2. Diagnosis of NP/OP samples from patients of the Hospital of the University of Pennsylvania (HUP) with COVID-19 symptoms using LEAD.

NP/OP Sample ID	COVID-19 Status	RT-PCR	LEAD
**VTMSET-07**	negative	-	+
**VTMSET-21**	negative	-	-
**VTMSET-35**	negative	-	-
**VTMSET-46**	negative	-	-
**VTMSET-55**	negative	-	-
**VTMSET-57**	negative	-	-
**VTMSET-58**	negative	-	-
**VTMSET-51**	negative	-	+
**VTMSET-59**	negative	-	-
**VTMSET-236**	negative	-	-
**VTMSET-237**	negative	-	-
**VTMSET-238**	negative	-	-
**VTMSET-239**	negative	-	-
**VTMSET-240**	negative	-	-
**VTMSET-241**	negative	-	-
**VTMSET-242**	negative	-	-
**VTMSET-243**	negative	-	-
**VTMSET-244**	negative	-	-
**VTMSET-245**	negative	-	-
**VTMSET-246**	negative	-	-
**VTMSET-247**	negative	-	-
**VTMSET-248**	negative	-	-
**VTMSET-249**	negative	-	-
**VTMSET-250**	negative	-	-
**VTMSET-251**	negative	-	+
**VTMSET-252**	negative	-	-
**VTMSET-253**	negative	-	-
**VTMSET-254**	negative	-	-
**VTMSET-255**	negative	-	-
**VTMSET-256**	negative	-	+
**VTMSET-257**	negative	-	-
**VTMSET-258**	negative	-	+
**VTMSET-259**	negative	-	-
**VTMSET-260**	negative	-	-

**VTMSET-261**	negative	-	-
**VTMSET-262**	negative	-	-
**VTMSET-263**	negative	-	-
**VTMSET-264**	negative	-	-
**VTMSET-265**	negative	-	-
**VTMSET-266**	negative	-	+
**VTMSET-267**	negative	-	-
**VTMSET-268**	negative	-	-
**VTMSET-269**	negative	-	-
**VTMSET-270**	negative	-	+
**VTMSET-271**	negative	-	-
**VTMSET-272**	negative	-	-
**VTMSET-273**	negative	-	-
**VTMSET-274**	negative	-	-
**VTMSET-275**	negative	-	-
**VTMSET-276**	negative	-	-
**VTMSET-04**	positive	+	+
**VTMSET-06**	positive	+	+
**VTMSET-33**	positive	+	+
**VTMSET-59**	positive	+	+
**VTMSET-60**	positive	+	+
**VTMSET-56**	positive	+	+
**VTMSET-53**	positive	+	+
**VTMSET-26**	positive	+	-
**VTMSET-40**	positive	+	+
**VTMSET-25**	positive	+	-
**1**	positive	+	+
**2**	positive	+	+
**3**	positive	+	+
**4**	positive	+	+
**5**	positive	+	+
**7**	positive	+	+
**27**	positive	+	+
**28**	positive	+	+
**29**	positive	+	+
**30**	positive	+	+
**31**	positive	+	+

**32**	positive	+	+
**33**	positive	+	+
**34**	positive	+	+
**35**	positive	+	+
**36**	positive	+	+
**37**	positive	+	+
**38**	positive	+	+
**39**	positive	+	-
**40**	positive	+	+
**43**	positive	+	+
**44**	positive	+	+
**45**	positive	+	+
**46**	positive	+	-
**50**	positive	+	+
**51**	positive	+	+
**52**	positive	+	+
**53**	positive	+	+
**54**	positive	+	+
**59**	positive	+	-
**60**	positive	+	+
**61**	positive	+	+
**62**	positive	+	-
**63**	positive	+	+
**64**	positive	+	+
**65**	positive	+	+
**66**	positive	+	+
**68**	positive	+	+
**72**	positive	+	+
**74**	positive	+	+
**76**	positive	+	+
**77**	positive	+	+

Table S3. Diagnosis of saliva samples from patients of the Hospital of the University of Pennsylvania (HUP) with COVID-19 symptoms using LEAD.

Saliva sample ID	COVID-19 status	RT-PCR	LEAD
3	Positive	+	+
33	Positive	+	+
90	Positive	+	+
9	Negative	-	-
14	Negative	-	-
21	Negative	-	-
24	Negative	-	-
41	Negative	-	-
43	Negative	-	-
44	Negative	-	-

SI References

1. L. F. de Lima, E. A. Pereira, M. Ferreira, Electrochemical sensor for propylparaben using hybrid Layer-by-Layer films composed of gold nanoparticles, poly(ethylene imine) and nickel(II) phthalocyanine tetrasulfonate. *Sensors Actuators, B Chem.* **310**, 1–8 (2020).
2. T. Chaibun, *et al.*, Rapid electrochemical detection of coronavirus SARS-CoV-2. *Nat. Commun.* **12**, 1–10 (2021).
3. A. Yakoh, *et al.*, Paper-based electrochemical biosensor for diagnosing COVID-19: Detection of SARS-CoV-2 antibodies and antigen. *Biosens. Bioelectron.* **176**, 112912 (2021).
4. L. Fabiani, *et al.*, Magnetic beads combined with carbon black-based screen-printed electrodes for COVID-19: A reliable and miniaturized electrochemical immunosensor for SARS-CoV-2 detection in saliva. *Biosens. Bioelectron.* **171** (2021).
5. H. Zhao, *et al.*, Ultrasensitive supersandwich-type electrochemical sensor for SARS-CoV-2 from the infected COVID-19 patients using a smartphone. *Sensors Actuators, B Chem.* **327**, 128899 (2021).
6. S. Eissa, M. Zourob, Development of a Low-Cost Cotton-Tipped Electrochemical Immunosensor for the Detection of SARS-CoV-2. *Anal. Chem.* (2021) https:/doi.org/10.1021/acs.analchem.0c04719.
7. A. Raziq, *et al.*, Development of a portable MIP-based electrochemical sensor for detection of SARS-CoV-2 antigen. *Biosens. Bioelectron.* **178** (2021).
8. Marcelo D.T. Torres, William R. de Araujo, Lucas F. de Lima, André L. Ferreira, Fuente-Nunez, Matter. *Matter*, 1–33 (2021).
9. G. Seo, *et al.*, Rapid Detection of COVID-19 Causative Virus (SARS-CoV-2) in Human Nasopharyngeal Swab Specimens Using Field-Effect Transistor-Based Biosensor. *ACS Nano* **14**, 5135–5142 (2020).
